# Complete chloroplast genome of *Vincetoxicum hainanense* (Apocynaceae: Asclepiadoideae), an endangered liana endemic to China

**DOI:** 10.1080/23802359.2019.1674714

**Published:** 2019-10-16

**Authors:** Wujian Xiong, Shiou Yih Lee, Panpan Liu, Wenhui You, Wenbo Liao

**Affiliations:** a Shanghai Key Lab for Urban Ecological Processes and Eco-Restoration, School of Ecological and Environmental Sciences, East China Normal University, Shanghai, PR China;; b State Key Laboratory of Biocontrol and Guangdong Provincial Key Laboratory of Plant Resources, School of Life Sciences, Sun Yat-sen University, Guangzhou, PR China;; c Zhongshan State-owned Forest Resources Protected Center, Zhongshan, PR China

**Keywords:** Apocynaceae, genomic resources, Illumina sequencing, phylogenetic analysis, *Vincetoxicum hainanense*

## Abstract

*Vincetoxicum hainanense* is an endangered liana species endemic to China. Habitat destruction coupled with difficulties in natural pollination has reduced its population size over time. As present studies have focussed more on breeding programmes instead of molecular aspects, here we reported on the complete chloroplast (cp) genome sequence of *V. hainanense*. The cp genome is 161,280 bp in size and includes two inverted repeat (IR) regions of 24,884 bp each, which is separated by a large-single copy (LSC) region of 92,084 bp and a small-single copy (SSC) region of 19,428 bp. A total of 131 genes were predicted, including 37 tRNA, 8 rRNA, and 86 protein-coding genes. Phylogenetic analysis showed that *V. hainanense* is clustered with other Apocynaceae species and sister to *Biondia chinensis*.

*Vincetoxicum hainanense* (Chun and Tsiang) Meve, H.H. Kong and Liede (synonym *Merrillanthus hainanensis*), a liana from the family Apocynaceae, is an endangered species endemic to China that is only confined to limited regions in Guangdong, and Hainan provinces (Chun and Tsiang 1941; Liede and Meve 2018). The liana grows in sandy soils, close to water source and has important ecological and ornamental values. While the liana is highly dependent to specific insects to pollinate in the wild (Liao et al. 2019); human activities and habitat destruction have threatened the survival of wild *V. hainanense*, resulted to reduction in population size (Miao et al. 2019). Although the China Plant Red Data Book has regarded this species as ‘Endangered’ (Fu and Zeng 1992), present studies have focussed more on breeding programmes instead of molecular aspects. In order to aid in conserving this valuable species, we assembled its complete chloroplast (cp) genome using Illumina sequencing technology, with the aim to provide useful genetic information on this endangered yet understudied species.

Total genomic DNA was extracted from *V. hainanense* leaves planted in the greenhouse of Sun Yat-sen University (SYSU), previously a wilding collected from Tianxin Forest Park, Zhongshan city, Guangdong province of China (N22°24′39′′, E113°28′21′′). Voucher specimen was deposited at the Herbarium of SYSU, Guangzhou, China, under the voucher collection number ZS-015. Next-generation sequencing was carried out using an Illumina Novaseq platform and the resultant clean reads were filtered and assembled into contigs using the GetOrganelle pipeline (http://github.com/Kinggerm/GetOrganelle), with SPAdes version 3.10.1 (http://cab.spbu.ru/software/spades/) as assembler (Bankevich et al. 2012). The complete cp genome was annotated using GeSeq (Tillich et al. 2017), and deposited in the NCBI GenBank under the accession number MN395661.

The complete cp genome of *V. hainanense* was 161,280 bp in size, which includes a large-single copy (LSC; 92,084 bp) region, a small-single copy (SSC; 19,428 bp) region, and a pair of inverted repeat (IR; 24,884 bp each) regions. The circular genome contained 131 genes, including 86 protein-coding genes, 8 rRNA genes, and 37 tRNA genes. The overall GC content was 37.67%.

For phylogenetic analysis, a maximum-likelihood (ML) phylogenetic tree was constructed with 1000 bootstrap replicates using RAxML programme (Stamatakis 2014). A subset of 13 Apocynaceae species was included, with *Gentiana officinalis* (Gentianaceae) as outgroup. The ML analysis showed that *V. hainanense* is clustered with other Apocynaceae species and sister to *Biondia chinensis* under a strong bootstrap (100%) (Figure 1). This finding could serve as valuable genomic resources in future genetic-based researches for this endangered plant species.

**Figure 1.**
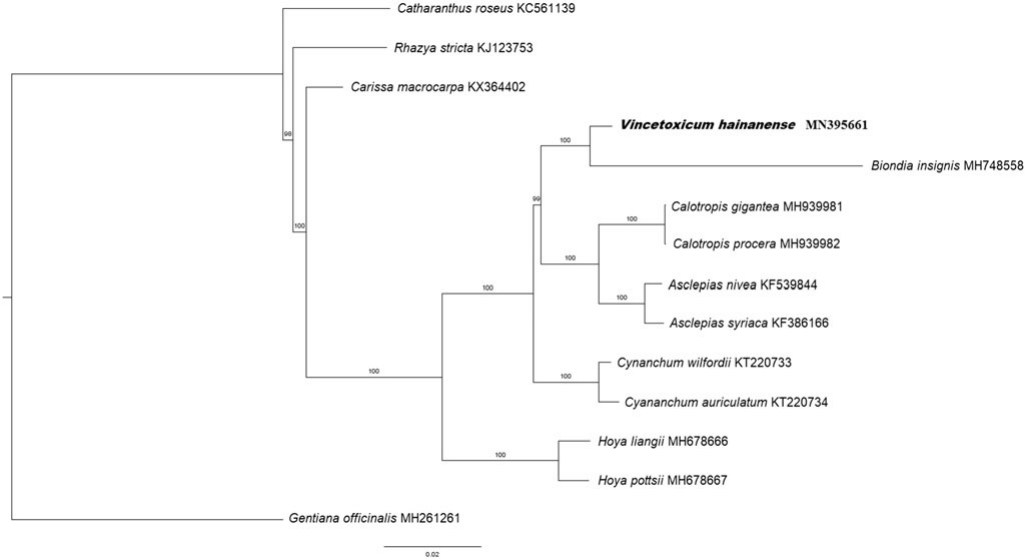
Maximum likelihood tree based on the complete chloroplast genome sequences of 13 species from the family Apocynaceae, with *Gentiana officinalis* (Gentianaceae) as outgroup. Shown next to the nodes are bootstrap support values based on 1000 replicates.

## References

[CIT0001] Bankevich A, Nurk S, Antipov D, Gurevich AA, Dvorkin M, Kulikov AS, Lesin VM, Nikolenko SI, Pham S, Prjibelski AD, et al. 2012 SPAdes: a new genome assembly algorithm and its applications to single-cell sequencing. J Comput Biol. 19(5):455–477. 22506599 10.1089/cmb.2012.0021 PMC3342519

[CIT0002] Chun W, Tsiang Y 1941 *Merrillanthus hainanensis. *Sunyatsenia. 6:107–108.

[CIT0003] Fu L, Zeng X 1992 China plant red data book: rare and endangered plants. Vol. 1 Beijing, China: Science Press; p. 454–455.

[CIT0004] Liao H, Feng L, Xiong W, Jiang Q, Zhang X, Ye F, Liao W 2019 Phenological and breeding characteristics of *Merrillanthus hainanensis* (*Apocynaceae, Asclepiadaceae*). Acta Sci Nat Univ Sunyatseni. 58:48–53.

[CIT0005] Liede SS, Meve U 2018 *Vincetoxicum* (*Apocynaceae-Asclepiadoideae*) expanded to include *Tylophora* and allies. Phytotaxa. 369:129–184.

[CIT0006] Miao S, Huang H, Tao W, Qin X, Chen W, Dai W 2019 Research progress on the wild key national protected species *Merrillanthus hainanensis*. Chin Wild Plant Resour. 38:52–55.

[CIT0007] Stamatakis A 2014 RAxML version 8: a tool for phylogenetic analysis and post-analysis of large phylogenies. Bioinformatics. 30(9):1312–1313. 24451623 10.1093/bioinformatics/btu033 PMC3998144

[CIT0008] Tillich M, Lehwark P, Pellizzer T, Ulbricht-Jones ES, Fischer A, Bock R, Greiner S 2017 GeSeq – versatile and accurate annotation of organelle genomes. Nucleic Acids Res. 45(W1):W6–W11. 28486635 10.1093/nar/gkx391 PMC5570176

